# Camouflage strategies interfere differently with observer search images

**DOI:** 10.1098/rspb.2018.1386

**Published:** 2018-09-05

**Authors:** Jolyon Troscianko, John Skelhorn, Martin Stevens

**Affiliations:** 1 Centre for Ecology and Conservation, College of Life and Environmental Sciences, University of Exeter, Penryn Campus, Penryn, Cornwall TR10 9FE, UK; 2 Centre for Behaviour and Evolution, Institute of Neuroscience, Newcastle University, Henry Wellcome Building, Framlington Place, Newcastle upon Tyne NE2 4HH, UK

**Keywords:** camouflage, search image, apostatic selection, learning, vision, cognition

## Abstract

Numerous animals rely on camouflage for defence. Substantial past work has identified the presence of multiple strategies for concealment, and tested the mechanisms underpinning how they work. These include background matching, D-RUP coloration to destroy target edges, and distractive markings that may divert attention from key target features. Despite considerable progress, work has focused on how camouflage types prevent initial detection by naive observers. However, predators will often encounter multiple targets over time, providing the opportunity to learn or focus attention through search images. At present, we know almost nothing about how camouflage types facilitate or hinder predator performance over repeated encounters. Here, we use experiments with human subjects searching for targets on touch screens with different camouflage strategies, and control the experience that subjects have with target types. We show that different camouflage strategies affect how subjects improve in detecting targets with repeated encounters, and how performance in detection of one camouflage type depends on experience of other strategies. In particular, disruptive coloration is effective at preventing improvements in camouflage breaking during search image formation, and experience with one camouflage type (distraction) can decrease the ability of subjects to switch to and from search images for new camouflage types (disruption). Our study is, to our knowledge, the first to show how the success of camouflage strategies depends on how they prevent initial and successive detection, and on predator experience of other strategies. This has implications for the evolution of prey phenotypes, how we assess the efficacy of defences, and predator–prey dynamics.

## 1. Introduction

Animal coloration is a long-standing test-bed for research into evolution [1], and camouflage in particular provides the most intuitive link between an animal's phenotype and its survival [2]. Aside from being a key paradigm to study natural selection and animal coloration, camouflage has also attracted attention from scientists interested in visual perception, cognition and computer vision [3,4]. The mechanisms by which different types of camouflage evade predator perception have attracted considerable attention in recent years [5–7], and there has been much testing of how different camouflage strategies prevent detection by subjects naive to the targets (e.g. [8–11]).

A number of distinct camouflage strategies have been identified [6,12,13], the most intuitive being ‘background-matching’ (BM), where prey resemble the general coloration and pattern of the background [14,15]. A distinct approach is ‘disruptive coloration’, where high contrast markings break up the outline of an animal's body while other markings blend into the background, destroying information about true form [8,12,13]. Disruptive coloration has been suggested to occur in numerous terrestrial and aquatic animals, from fishes and frogs to moths and birds [12,16]. However, most work has focused on testing whether disruption works in artificial systems and only a handful of objective tests or quantification of disruption in real animals exist, such as in frogs [17], marine isopods [18], moths [19] and birds [20]. More controversially, conspicuous ‘distractive’ markings may direct the attention of the predator away from the prey's key features such as body outlines or limbs, or destroy detection of outlines through contour inhibition [6,7,13]. Examples of putative distractive markings in nature include the bright white ‘comma’ marking on the underside of the wings of the comma butterfly (*Polygonia c-album*), and white markings on the silver Y moth (*Autographa gamma*). However, this idea is controversial ([21,22]; see Discussion). Despite much progress, previous work on camouflage strategies focuses almost exclusively on how camouflage types have evolved to exploit predator sensory processes, and fails to consider that they may also exploit predators' attentional or cognitive processes [23].

A major gap in our understanding of camouflage strategies is that little is known regarding how predators perform in detecting camouflaged prey over repeated encounters, particularly when predators switch between prey that use different strategies. This is important because predator experience and cognition has the potential to dramatically shape the evolution of animal camouflage through a variety of processes (see below; [23,24]). Broadly, detection could improve over repeated encounters in two ways: discrimination learning plays an important role when learning to detect prey in a novel cryptic context [25], and once learning is complete, predators can form short-term search images over successive encounters with a single familiar prey type by selectively attending to salient prey features [26]. However, by forming a search image for one prey type, predators correspondingly suffer a reduction in their ability to find prey of other phenotypes [24,27]. There is now a body of research demonstrating that species learn about, and form search images for camouflaged prey [24,28–30], and some evidence that predator experience influences the efficacy of camouflage strategies and features [10,31]. Yet despite the fact that aspects of prey appearance, such as conspicuousness, are widely known to influence the speed of discrimination learning in other contexts [32], and how search images can reduce detection times [26], no attempt has been made to investigate how different camouflage types influence discrimination learning and search image formation. Fundamentally, it is currently unknown if and how the efficacy of one camouflage type is influenced by the presence of other camouflage types and experience of them.

A lack of consideration of predator experience and cognition in the efficacy and evolution of camouflage strategies limits our understanding of the factors responsible for the diversity existing in animal phenotypes, both within and between species. For example, predator behaviour related to changes in attention, learning and experience can promote diversity through mechanisms such as negative frequency-dependent (apostatic) selection [24,33–35]. Through repeated encounters with a given prey phenotype, predator attention is thought to focus on one search image for that prey appearance [24,36]. Importantly, while searching for one prey appearance, the predator's ability to find prey with a different appearance can be inhibited, giving a disproportionate advantage to uncommon prey types [27,34,37,38]. This is widely thought to drive many of the remarkable prey polymorphisms that exist in nature (e.g. [28,39]). Tantalizingly, observer experience has also been found to interact with different types of camouflage, with some strategies defeated more readily than others by observers over time/with greater experience [10,31]. For example, over repeated interactions with distractive markings, predators become faster at finding them compared to other prey types lacking such prominent features [31], but in other contexts, relative performance improvements towards distractive markings can be lower [10]. Nevertheless, the reasons for changes in predator performance in breaking different camouflage strategies with exposure remain unknown (e.g. learning, familiarization, attention), and no studies have investigated how different camouflage strategies could interact with predator cognition and search image effects. This is important because in nature, there is often considerable variation in camouflage strategy and appearance both within and among prey species. If the value of one camouflage strategy is dependent on the presence of other camouflage strategies, and of predator experience, the evolution, maintenance and adaptive value of camouflage forms in nature can only be fully understood in the wider context of the predator–prey community, rather than simply in terms of camouflage value in isolated encounters (which is where work has to date focused).

In this study, we test how repeated encounters with different camouflage strategies affects predator performance. We developed a serial detection task to determine how repeated experience of targets with one camouflage strategy affects an observer's ability to switch to detecting targets with another camouflage strategy. Our ultimate aim is to determine whether some camouflage strategies facilitate or hinder predator performance over repeated interactions, and to what extent, predator experience and search image formation associated with one camouflage type hinders switching to find a new camouflage type.

## 2. Methods

**Figure 1.**
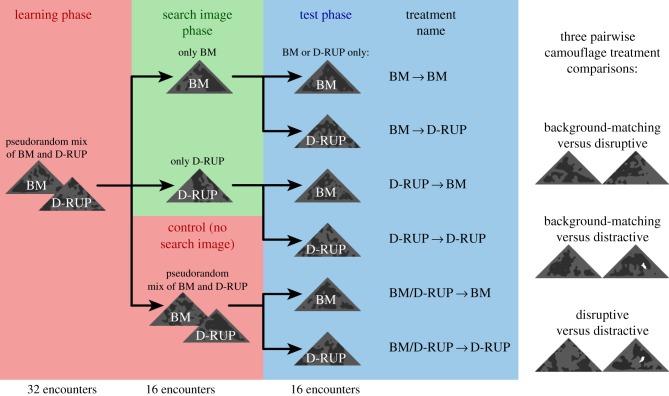
Schematic showing the experimental design in the BM versus D-RUP camouflage comparison. Each participant was allocated to one of the six treatment types shown. All participants initially received 32 pseudorandom encounters with both types of camouflage treatment (pink), with one prey shown on each screen, ensuring all participants had experience of finding both prey types. Participants then moved to one of three blocks, either receiving a solid run of only one camouflage type (search image phase; green) or continuing to receive a pseudorandom mix of treatments over 16 encounters (pink). Finally, all participants received a solid run of one camouflage type over 16 encounters (blue). This experimental design was repeated three times in pairwise comparisons between the three camouflage types (shown on the right).

**Figure 2.**
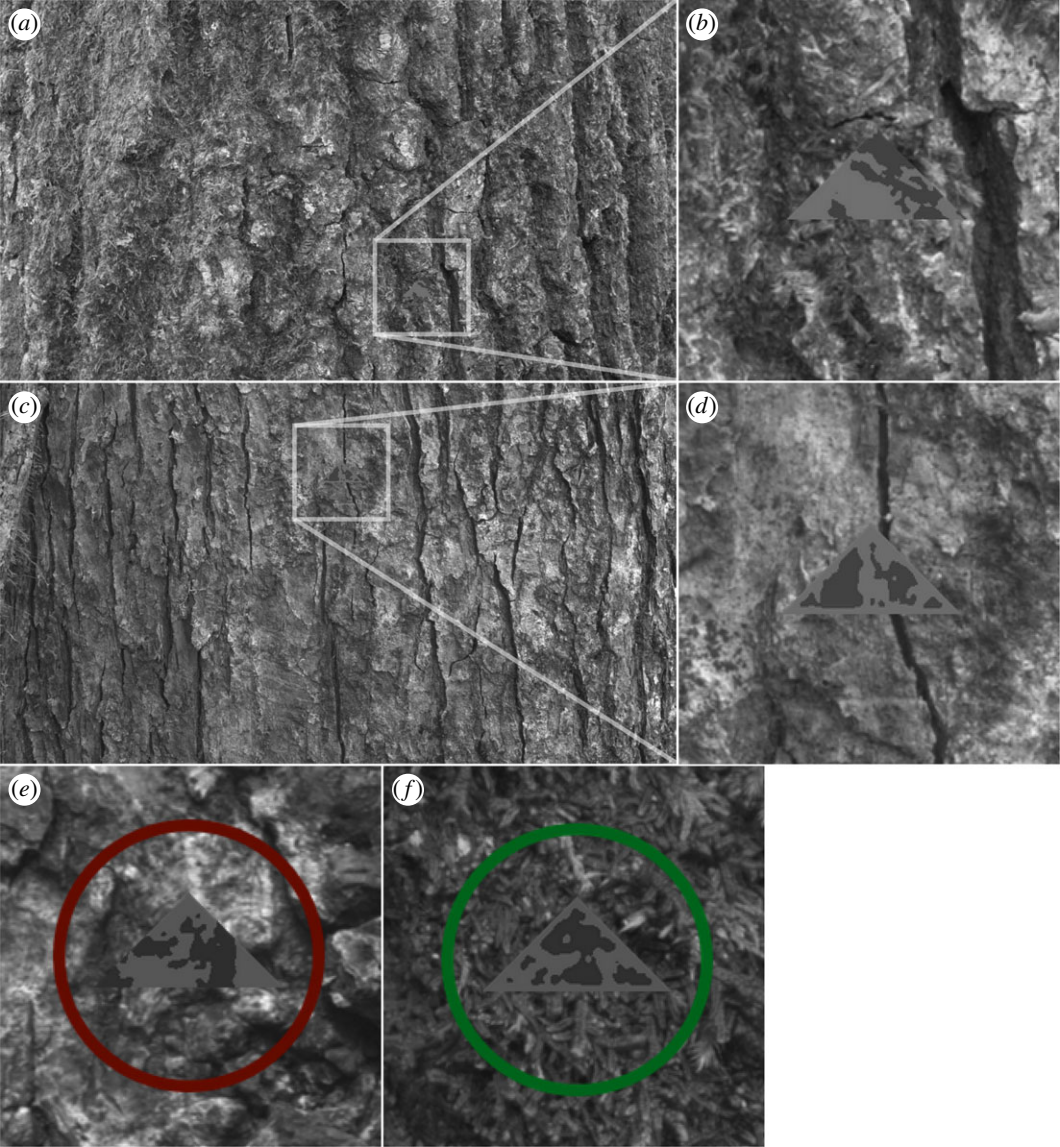
Sample screen-shots of the game showing a disruptive target (*a*) as seen by participants, with close-up illustration (*b*); a BM target (*c*), with close-up (*d*). When participants failed to find the target during the learning phase, a red circle (*e*) was presented around the target for 1 s. Successful captures were encircled in green (*f*).

Prey were created using patterns taken from the natural background against which they were presented (see below) with one of the three treatments: BM, where patterns within the prey match the background but did not reach to its edges (e.g. [8]), ‘disruptive’ (D-RUP), where the prey's patterns intersected its edges, and ‘distractive’ (D-RAC), where a salient high contrast marking was placed inside the prey's outline (e.g. [31]). In creating the D-RUP targets, we followed numerous past studies (e.g. [8,10,40]) in having targets that matched the backgrounds but with the stipulation that at least some marking components intersected the body outline in a disruptive manner. In making the BM targets, we followed the same method but stipulated that no markings should intersect the body outline. This also follows a range of past work [8,10,40], and also avoids the pitfalls of simply shifting markings inwards from the target outline, which can create inside edges running alongside the target margins and increase the density of markings internally (see [8,40]). It should be noted that in nature, these two strategies need not be independent, and that BM prey may also possess markings that intersect the body outlines, even just by chance. However, here, as in past work, our aim was to test the effects of these conceptually distinct strategies on search image formation and target switching. In order to test for search image effects of general camouflage type rather than for specific prey appearance, the prey in our task were all individually unique [10,41]. BM and D-RAC prey were either presented as light-on-dark or dark-on-light. Even though no two prey appearances were identical, previous research has demonstrated that powerful search image effects can form for continuously variable prey [28]. A total of 360 participants were recruited from the University of Exeter, Penryn Campus to play a touchscreen computer experiment, with 20 individuals per treatment. Artificial triangular prey (126 by 64 pixels) were generated from the natural tree bark background images they were shown against following the methods described in previous studies [10,41]. Samples of the prey are shown in figure 1 and the set-up is shown in figure 2.

The game was programmed in custom HTML5/Javascript code and presented on an Acer T272HL LCD touch-screen monitor with a display area of 600 mm by 338 mm, and resolution of 1920 by 1080 pixels. Each participant received 64 slides in total, and encountered two types of camouflage (BM and D-RUP, BM and D-RAC, or D-RUP and D-RAC). In each slide, one prey item was presented in a random location on the screen and participants were asked to click the prey as soon as they saw them. If the participant clicked on the prey within 30 s, the capture time was recorded to the nearest millisecond and they progressed to the next slide. Each trial consisted of three sections; in the first 32 slides, both prey types were presented in a pseudorandom order, ensuring the participant received no more than two encounters with each prey type sequentially. This ‘*training phase*’ is essential to ensure each participant had controlled and balanced prior experience of both prey types without forming a search image for either [24]. The length of this training phase (i.e. 32 encounters) was based on pilot data which indicated that participants failed to increase in their detection times any further after approximately 30 encounters with these prey (R. Troscianko, J Skelhorn, M Stevens 2014–2015, unpublished data). Next, the participant received a 16-long run of prey with only one camouflage type (the ‘*search image phase*’), while a control group continued to receive the pseudorandom mix. Finally, all participants received the ‘*test phase*’ where each participant received a 16-long run of only one camouflage type (figure 1).

In each trial, there was a 30 s timeout, after which the participant was moved on to the next slide. During the training phase, a ‘hit’ prey would have a green circle displayed around it for 2 s before the screen blanked and a new slide was shown. If the participant reached the 30 s timeout during the training phase, a red circle was displayed around the prey for 2 s to ensure the participant saw the prey that they missed, and controlling the training exposures. During the search image and test phases, the slide (whether prey were hit or missed) progressed immediately without circles being shown. The participants and experimenter were blind to the treatment type of all trials. At the start of each trial, the participant clicked a box confirming that they were happy for their anonymous data to be used for scientific purposes. No personal identifying data were collected. Twenty participants were recruited for each treatment combination, resulting in 120 participants per pairwise camouflage comparison, and a total of 23 040 unique prey presentations.

### (a) Statistics

**Figure 3.**
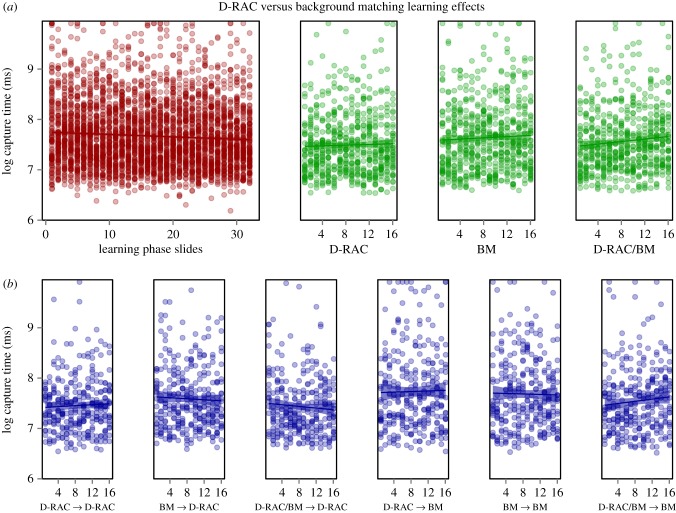
Experiment ‘B’ (BM versus D-RAC) demonstrated an effect of subject detection performance between trial phases. The learning phase is shown in red (*a*, left), the search image phases in green (*a*, right), and the test phases in blue (*b*).

All statistical tests were performed in R v. 3.0.2 [42]. For each experiment, a full mixed linear model was specified using the lme4 package [43] with logged capture time as a response. Each model contained the target's X and Y screen coordinates, fitted as a quadratic with an interaction between the two variables to account for the increased time taken to capture targets near the edge of the screen, or corners of the screen (interaction). Participant identity (ID) and background image ID were fitted as random effects to control for pseudoreplication. Additional fixed effects were whether the target was light-on-dark, or dark-on-light (inverted), slide number within each phase (to investigate learning differences that occurred within each phase), and the phase treatment (i.e. ‘learning phase’, one of the three ‘search image’ phases, or one of the six ‘test’ phases). Phase treatment was therefore a factor with 10 levels: learning, searchA, searchAB, searchB, testA–A, testA–B, testAB–A, testAB–B, testB–A, testB–B (where A and B are the relevant camouflage treatments in each of the experiments). The full model was fitted with two-way interactions between these three fixed effects. We used likelihood ratio tests (LRTs) to determine whether certain variables and interactions in the full model were redundant. Specifically, we tested for the redundancy of the interaction between slide number and phase, the interaction between X and Y screen coordinates, and the inclusion of the ‘inverted’ factor. LRT and Akaike information criterion-based model selection procedures were in agreement. Planned comparisons between treatments were then investigated in the simplified models using LMERtest. The R-code used to analyse the data are included as the electronic supplementary material. Planned comparisons between treatments were then investigated in the simplified models using LMERtest. In all three experiments, the ‘inverted’ variable was not found to affect capture times, so was removed from the model. In each model, we first tested for within-phase difference in capture times; however, it was only in experiment B, where this interaction between slide number and phase was significant (LRT 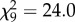, *p* = 0.004; figure 3 shows learning rates).

## 3. Results

In our experiment, human participants were tested on their ability to form and switch between search images for artificially generated prey presented against natural backgrounds on a touchscreen computer. Participants were first presented with a training sequence of two pseudorandomly assorted prey types to ensure each participant was equally experienced in finding both prey types (*learning phase*). Next, they were either given a straight sequential run of just one of the prey types to allow search image formation (*search image phase*), or they were in a control group that continued to receive pseudorandomly interspersed prey types, which previous research demonstrates prevents search image formation [30]. Finally, participants received a sequential run of just one prey type in a *test phase*, creating six different experimental learning treatments shown in figure 1. This pairwise design was used in three experiments that compared BM to D-RUP targets (experiment A), BM to D-RAC (experiment B), and D-RUP to D-RAC (experiment C). The results of planned pairwise statistical tests between experimental treatments are shown in table 1.

**Table 1.** Full statistical results for each of the three experiments and within and among participant capture time comparisons over the three experimental test phases. (An asterisk denotes a statistically significant test result.)

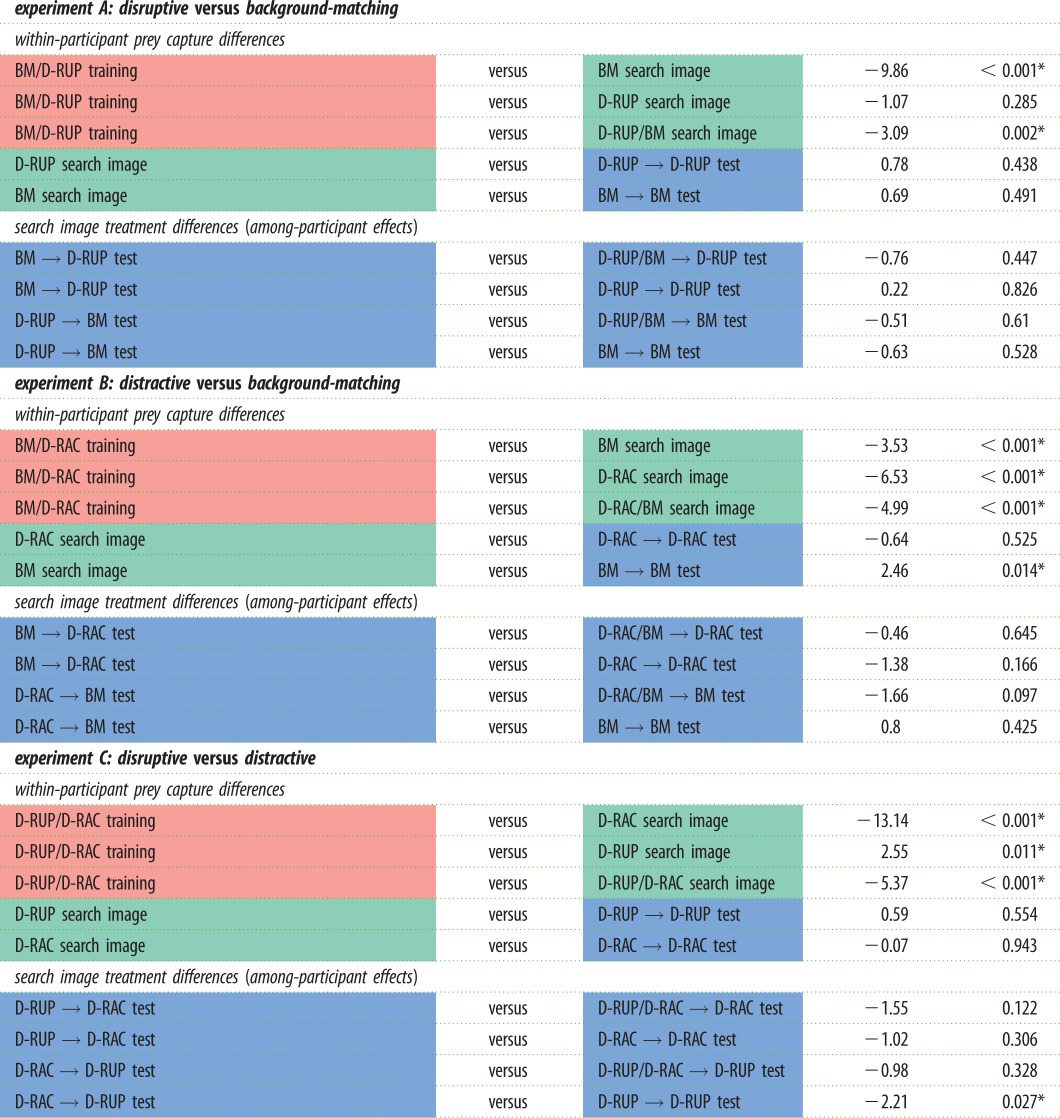

First, we investigated prey capture times in instances where individual participants switched treatments (labelled ‘within-participant prey capture differences' in table 1). Overall, there was a significant tendency for capture times to decrease between training (learning) and search image phases (all *p* ≤ 0.002), demonstrating improvement in performance as participants encountered more prey (even when moving from interspersed training to an interspersed ‘control’ search image phase). The only two exceptions to this rule are the two treatments where participants switched from training to a run of D-RUP prey. In this case, there was no significant difference in capture times (BM/D-RUP training versus D-RUP search image, *t* = −1.07, *p* = 0.285) or even a significant increase in capture times (D-RUP/D-RAC training versus D-RUP search image, *t* = 2.55, *p* = 0.011). Based on this finding, we also tested whether D-RUP prey interspersed with background matching prey could interfere with performance; we found no difference in performance between the D-RUP sequential search image phase and interspersed D-RUP/BM search image (control) phase (*t* = −1.51, *p* = 0.132). However, D-RUP prey interspersed with D-RAC prey did not interfere with participants' ability to improve their performance (D-RUP search image prey took significantly longer to find than D-RUP/D-RAC search image prey *t* = −5.87, *p* < 0.001). These results demonstrate that D-RUP prey can interfere with participant's ability to improve in their performance via a lack of search image formation, and even result in worsening performance, and this can even happen when D-RUP prey are interspersed with other prey types. There was no tendency for participants to improve their performance where they continued to receive continuous runs of the same treatment between search image and test phases (all *p*-values ≥ 0.438). The only exception to this rule were participants in experiment B who received BM search image phase and BM → BM test phase, capturing prey significantly slower in the test phase (*t* = 2.46, *p* = 0.014). This effect is probably owing to fatigue in the participants.

Finally, we tested whether the formation of a search image for one camouflage strategy affected the ability of participants to switch to finding prey with a different camouflage strategy (comparing *search image* and *test phase* treatments). These tests compare performance among participants, who were only able to experience one search image and test treatment combination (labelled ‘search image treatment differences (among participant effects)’ in table 1). Only one treatment combination was found to show a significant effect of search image formation compared to controls; participants who switched from D-RAC to D-RUP prey D-RAC → D-RUP) had significantly longer capture times than those who received only D-RUP (D-RUP → D-RUP: *t* = 2.21, *p* = 0.027). This suggests that forming a search image for D-RAC targets makes it more difficult than expected to switch to finding D-RUP targets. All other treatments resulted in non-significant differences (*p* ≥ 0.097).

## 4. Discussion

Using a series of novel and carefully controlled experiments, we have shown, to our knowledge, for the first time how different camouflage strategies can facilitate or interfere with human learning and search image formation. Animals are thought to have limited attention with which to search for prey while foraging [44], and over successive encounters with one prey type, predators can form a temporary search image which, while improving success in finding one prey type, interferes with their ability to switch to finding new prey types [27,37,38]. Our work here shows that search image effects are influenced by conceptually distinct strategies of camouflage. Most strikingly, we find that observer performance is most negatively affected by disruptive coloration, which often hinders improvement in performance with experience. In addition, we also demonstrate a search image interference effect between camouflage strategies, with switching from distractive to disruptive strategies most costly to performance.

Disruptive coloration is an effective camouflage strategy hindering detection by breaking up an animal's outline [12], and numerous previous studies using artificial stimuli have demonstrated that prey with disruptive markings and greater levels of disruptiveness are more effective at increasing capture times compared to other forms of camouflage (e.g. [8,9,11,41,45–47]). However, such work has mostly focused on how disruption prevents initial detection (but see [10,31]). Our results show that disruptive coloration can be highly effective at blocking improvements in camouflage breaking in observers over time/experience. Therefore, the success of disruptive coloration in both artificial and likely natural systems can be attributed both to preventing initial detection and through impeding the ability of predators to improve performance with experience. This effect is likely to contribute to the apparent abundance of disruptive patterns in nature [12,16]. However, the question of why disruptive coloration seems so effective in preventing capture even as participants gain experience remains to be tested. One option is that it removes information corresponding to body edges and shapes, which may otherwise provide cues for both initial detection and information that could be learnt or used to prime attention by observers.

Intriguingly, we also found that having disruptive prey interspersed with background matching prey hindered the participants' ability to learn to find any of the prey faster. This was perhaps owing to interference with the participants’ ability to form search images for the overall prey outline (which is intact in background matching prey). This experience-interference effect of disruptive prey was not found when interspersed with the more easily learnt distractive prey, which had both an intact outline and a high contrast (black or white) marking. High-contrast prey have previously been shown to be easier to learn to find over successive encounters than lower contrast prey [10], and the distractive marking placed on prey would have effectively increased target contrast. We cannot therefore be certain whether the randomly shaped distractive spot or the general increase in prey contrast was the attentional cue used by participants when forming a search image. However, it is clear that the presence of disruptive species in prey communities could potentially enhance the survival of species with other forms of camouflage.

Distractive markings are often thought to function by attracting the gaze or attention of the predator away from key features (such as the prey's outline) that would otherwise make it stand out to predators [6,7,13]. The concept of distractive markings is controversial, with studies in artificial systems sometimes reporting evidence for a distractive effect, which other studies have questioned, or conversely finding significant costs of possessing putative distractive markings [10,21,22,31,48,49]. However, in real animals, there exist various markings that are puzzling with their conspicuousness on an otherwise well-concealed body; most notably, the small bright markings found on the wings of some cryptic moths and butterflies; with the most common example being that of the comma butterfly. One study on this species reports support for the distractive concept, in that butterflies with the characteristic white wing marking were attacked less by captive birds than butterflies where the marking was painted over [50]. However, unfortunately, that experiment was not able to test distraction because the butterflies were presented in a small box in close proximity to the birds, and against a uniform brown background that the authors acknowledge made all the butterflies ‘fully visible’. That is, the butterflies were not camouflaged. Instead, the results are more likely explained by neophobia or avoidance of the bright marking itself. In a field component of that study, there was no effect of the comma marking. More work, especially in real animals under natural conditions, is needed to test whether such markings have this function in nature. Here, in line with previous findings using similar set-ups [10,31], this study demonstrates that distractive markings facilitate improved capture rates over time, and offer no protection over and above BM. This improved performance effect implies participants are (consciously or unconsciously) using attentional filtering and forming a search image for distractive prey. When these participants switched to receiving only disruptive prey, they performed worse than participants who had only ever encountered disruptive prey. The question of why this occurred requires further work, but it is probable that distractive markings provide salient and reliable search features [26] for locating targets which overshadow other prey features, including the body outline. In addition, because disruption effectively destroys the edges of targets and key features, it may reduce the available information that subjects can use over time to guide their search behaviour. As a result, it takes longer for observer performance to recover when switching to disruptive targets.

**Figure 4.**
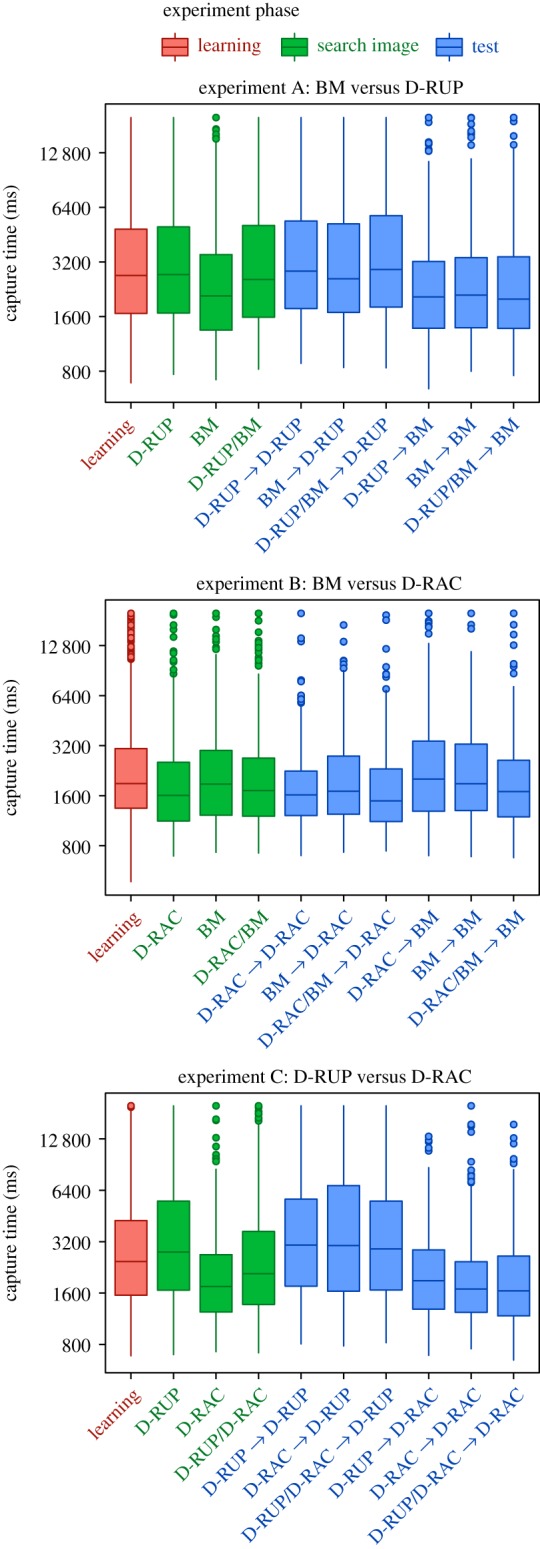
Capture times in the three experiments, showing nested experiment phases, i.e. the ‘learning’ phase shows all participants' (120 in each experiment) capture times across the first 32 slides, the ‘search image’ phase shows the three search image combinations, each with 16 slides and 40 participants, the ‘test’ phase shows each experimental treatment with the final 16 slides and 20 participants. ‘BM’ is background matching, ‘D-RUP’ is disruptive and ‘D-RAC’ is distractive prey types.

The search rate hypothesis has been suggested as an alternative mechanism for changes in detection of prey over time that does not depend on forming a search image [51]. Here, predators could change their search rates depending on prey conspicuousness; when easily found prey are most abundant the predator should use a fast search rate, whereas a slower search rate may be more effective with highly cryptic prey. The nature of our camouflage strategy comparisons made it impossible to simultaneously make each prey camouflage type equally difficult to detect (doing so would require changing some aspect of the prey that might influence learning rates, such as contrast, size or shape). While there were overall differences in prey conspicuousness dependent on camouflage type, there was considerable overlap in the capture times between these types (figure 4). It may therefore be impossible for us to entirely rule out search rate effects in this study. However, we believe this is less likely to explain our findings than the search image hypothesis because participants who were prevented from forming a search image (owing to receiving interspersed disruptive and distractive prey), and were then forced to find disruptive prey only, performed no differently from those who were switched from a distractive prey search image to disruptive prey. The search rate hypothesis would have predicted a decrease in capture times following a switch from no search image to disruptive prey only. In addition, search image but not search rate theory predicts improved performance in finding the same type of prey over time/with experience [52], which we often found here.

Camouflage is the most common anti-predator defence, with a range of different strategies found in nature. Predator learning has the potential to select for variation in camouflage strategies through frequency-dependent selection, and here, we have shown how predator performance with experience for one camouflage strategy can influence the effectiveness of other strategies. Our findings address a so-far neglected area of the value of different camouflage strategies, being that the overall value of a camouflage phenotype or strategy will often depend not only on how well it prevents initial detection from a naive observer, but also how the appearance of other sympatric prey interacts with predator search performance over time. Ultimately, these effects could considerably shape the evolution of animal coloration, including in the mechanics of intraspecific and interspecific frequency-dependent selection for broad types of camouflage strategies in prey communities that share predators. For example, considerable attention in evolutionary studies have explored the basis of prey polymorphisms in nature (e.g. [39,53]) and how predator cognition can shape the diversity and dynamics of virtual prey [28,38]. This work has illuminated our understanding of evolution, perception and behaviour, and predator–prey dynamics, yet has not considered fundamental issues related to camouflage type and efficacy. Our work here shows how camouflage appearances both among and between species may influence the value of concealment over time, providing important implications for patterns of prey appearance in natural populations. The implications may also spread to interactions within predator communities and the respective benefit of camouflage strategies over time. To date, work has focused on the value of camouflage strategies in isolation, but our study suggests that the benefit of one camouflage strategy may be influenced by the presence of other co-occurring strategies. For example, the benefit of disruptive coloration may be even higher when in the presence of distractive camouflage or prey with other salient markings. Ultimately, there is a need to understand the value of camouflage types in a wider context of both how their value changes with predator experience and the wider predator–prey community.

## Supplementary Material

Full data for each experiment

## Supplementary Material

R code

## Data accessibility

All data and R code for statistics are available as electronic supplementary material.
