# Supplementary material for: Camouflage strategies interfere differently with observer search images
Source: Proc Biol Sci. 2018 Sep 5;285(1886):20181386. doi: 10.1098/rspb.2018.1386 (PMC6158535; doi:10.1098/rspb.2018.1386)
Supplement: R code [file rspb20181386supp2.docx]

setwd("~/Documents/Work/Camouflage Learning/Experiment 2/Stats")

save.image()

library(ggplot2)

library(lme4)

library(lmerTest)

library(LMERConvenienceFunctions)

# ----------------------Data Import----------------------

aData <- read.csv("~/Documents/Work/Camouflage Learning/Experiment 2/Stats/Exp2A Disruptive BM data.csv")

bData <- read.csv("~/Documents/Work/Camouflage Learning/Experiment 2/Stats/Exp2B Distractive BM data.csv")

cData <- read.csv("~/Documents/Work/Camouflage Learning/Experiment 2/Stats/Exp2C Disruptive Distractive data.csv")

View(aData)

View(bData)

View(cData)

aData$Participant <- as.factor(aData$Participant)

bData$Participant <- as.factor(bData$Participant)

cData$Participant <- as.factor(cData$Participant)

aData$phaseSlide <- as.numeric(aData$phaseSlide)

bData$phaseSlide <- as.numeric(bData$phaseSlide)

cData$phaseSlide <- as.numeric(cData$phaseSlide)

aData$logTime <- log(aData$timeTo20)

bData$logTime <- log(bData$timeTo20)

cData$logTime <- log(cData$timeTo20)

qplot(phaseInc, logTime, data = aData, geom = "boxplot") + aes(fill = trialPhase)

qplot(phaseInc, logTime, data = bData, geom = "boxplot") + aes(fill = trialPhase)

qplot(phaseInc, logTime, data = cData, geom = "boxplot") + aes(fill = trialPhase)

#--------------------EXP2A DISRUPTIVE VS BACKGROUND MATCHING-----------------------

# "A" = Disruptive

# "B" = Background Matching

citation(package="base")

citation(package="lme4")

a1 <- lmer(logTime~ edgeDist + inverted + phaseSlide * phaseInc + (1|Participant) + (1|backgroundID), data=aData)

summary(a1)

mcp.fnc(a1) # looks ok, not perfect..

pamer.fnc(a1)

a2 <- lmer(logTime~ poly(TargetX,2) + poly(TargetY,2) + inverted + phaseSlide * phaseInc + (1|Participant) + (1|backgroundID), data=aData)

summary(a2)

pamer.fnc(a2)

anova(a1,a2) # plotting polynomial of target position is way better, massively significant and big AIC & BIC difference

# Plotting polynomials shows how it takes people onger to find targets near the edge of the screen

ggplot(aData, aes(x=TargetX, y=logTime)) +

geom_point(shape=1) + # Use hollow circles

stat_smooth(method = "lm", formula = y ~ poly(x, 2), size = 1) # Add polynomial curve

ggplot(aData, aes(x=TargetY, y=logTime)) +

geom_point(shape=1) + # Use hollow circles

stat_smooth(method = "lm", formula = y ~ poly(x, 2), size = 1) # Add polynomial curve

a3 <- lmer(logTime~ poly(TargetX,2) * poly(TargetY,2) + inverted + phaseSlide * phaseInc + (1|Participant) + (1|backgroundID), data=aData)

summary(a3)

pamer.fnc(a3)

anova(a3,a2) # the interaction between X & Y also improves the fit, explaining about 1.23% deviance.

# Over the edge dist value this is way better (10.7% deviance explained in a3 instead of 3.0% in a1)

# Remove learning over slides

a4 <- lmer(logTime~ poly(TargetX,2) * poly(TargetY,2) + inverted + phaseSlide + phaseInc + (1|Participant) + (1|backgroundID), data=aData)

summary(a4)

pamer.fnc(a4)

anova(a3,a4) # NS,

# There is no evidence for an interaction between slide number and trial phase (i.e. no difference in learning rates between phases, chi square =12.836 on 9 DF p = 0.1702)

a5 <- lmer(logTime~ poly(TargetX,2) * poly(TargetY,2) + phaseSlide + phaseInc + (1|Participant) + (1|backgroundID), data=aData)

summary(a5)

pamer.fnc(a5)

anova(a4,a5) # NS, implying "inverted" can be left out (Chi square = 0.146 on 1 DF, p = 0.7024)

# ----------------------EXP2A final disruptive vs BM model--------------------------

#Make planned comparisons between test treatments with moth treatment removed (incase it interacts with phaseInc)

# Relevel for testA-A

aData$phaseInc <- relevel(aData$phaseInc, ref = "testA-A")

a5 <- lmer(logTime~ poly(TargetX,2) * poly(TargetY,2) + phaseSlide + phaseInc + (1|Participant) + (1|backgroundID), data=aData)

summary(a5)

# Estimate Std. Error df t value Pr(>|t|)

# phaseInclearning -2.547e-03 4.521e-02 6.601e+03 -0.056 0.9551

# phaseIncsearchA -3.818e-02 4.924e-02 7.557e+03 -0.775 0.4381

# phaseIncsearchAB -1.054e-01 5.402e-02 5.489e+03 -1.952 0.0510 .

# phaseIncsearchB -3.310e-01 5.407e-02 5.493e+03 -6.122 9.90e-10 ***

# phaseInctestA-B -3.256e-01 6.109e-02 6.607e+03 -5.330 1.02e-07 ***

# phaseInctestAB-A -6.082e-02 6.205e-02 5.786e+03 -0.980 0.3270

# phaseInctestAB-B -3.573e-01 6.207e-02 5.771e+03 -5.757 9.02e-09 ***

# phaseInctestB-A -1.367e-02 6.209e-02 5.779e+03 -0.220 0.8258

# phaseInctestB-B -3.648e-01 6.207e-02 5.779e+03 -5.878 4.39e-09 ***

# lots of significant differences

# Relevel for testB-B

aData$phaseInc <- relevel(aData$phaseInc, ref = "testB-B")

a5 <- lmer(logTime~ poly(TargetX,2) * poly(TargetY,2) + phaseSlide + phaseInc + (1|Participant) + (1|backgroundID), data=aData)

summary(a5)

# phaseInctestA-A 3.648e-01 6.207e-02 5.779e+03 5.878 4.39e-09 ***

# phaseInclearning 3.623e-01 4.522e-02 6.636e+03 8.011 1.33e-15 ***

# phaseIncsearchA 3.267e-01 5.405e-02 5.513e+03 6.044 1.60e-09 ***

# phaseIncsearchAB 2.594e-01 5.410e-02 5.520e+03 4.795 1.67e-06 ***

# phaseIncsearchB 3.386e-02 4.921e-02 7.558e+03 0.688 0.4914

# phaseInctestA-B 3.923e-02 6.209e-02 5.790e+03 0.632 0.5275

# phaseInctestAB-A 3.040e-01 6.206e-02 5.802e+03 4.898 9.92e-07 ***

# phaseInctestAB-B 7.513e-03 6.212e-02 5.788e+03 0.121 0.9038

# phaseInctestB-A 3.512e-01 6.107e-02 6.631e+03 5.750 9.33e-09 ***

# Relevel for testAB-A

aData$phaseInc <- relevel(aData$phaseInc, ref = "testAB-A")

a5 <- lmer(logTime~ poly(TargetX,2) * poly(TargetY,2) + phaseSlide + phaseInc + (1|Participant) + (1|backgroundID), data=aData)

summary(a5)

# phaseInctestB-B -3.040e-01 6.206e-02 5.802e+03 -4.898 9.92e-07 ***

# phaseInctestA-A 6.082e-02 6.205e-02 5.786e+03 0.980 0.3270

# phaseInclearning 5.827e-02 4.517e-02 6.646e+03 1.290 0.1970

# phaseIncsearchA 2.264e-02 5.402e-02 5.523e+03 0.419 0.6752

# phaseIncsearchAB -4.462e-02 4.922e-02 7.560e+03 -0.907 0.3647

# phaseIncsearchB -2.702e-01 5.401e-02 5.531e+03 -5.002 5.85e-07 ***

# phaseInctestA-B -2.648e-01 6.207e-02 5.792e+03 -4.266 2.02e-05 ***

# phaseInctestAB-B -2.965e-01 6.103e-02 6.635e+03 -4.858 1.21e-06 ***

# phaseInctestB-A 4.716e-02 6.201e-02 5.807e+03 0.760 0.4470

# Relevel for testAB-B

aData$phaseInc <- relevel(aData$phaseInc, ref = "testAB-B")

a5 <- lmer(logTime~ poly(TargetX,2) * poly(TargetY,2) + phaseSlide + phaseInc + (1|Participant) + (1|backgroundID), data=aData)

summary(a5)

# phaseInctestAB-A 2.965e-01 6.103e-02 6.635e+03 4.858 1.21e-06 ***

# phaseInctestB-B -7.513e-03 6.212e-02 5.788e+03 -0.121 0.9038

# phaseInctestA-A 3.573e-01 6.207e-02 5.771e+03 5.757 9.02e-09 ***

# phaseInclearning 3.548e-01 4.523e-02 6.628e+03 7.845 4.88e-15 ***

# phaseIncsearchA 3.191e-01 5.406e-02 5.497e+03 5.903 3.78e-09 ***

# phaseIncsearchAB 2.519e-01 4.922e-02 7.559e+03 5.118 3.17e-07 ***

# phaseIncsearchB 2.635e-02 5.408e-02 5.516e+03 0.487 0.6261

# phaseInctestA-B 3.172e-02 6.210e-02 5.773e+03 0.511 0.6095

# phaseInctestB-A 3.437e-01 6.209e-02 5.804e+03 5.535 3.25e-08 ***

# Relevel for testA-B

aData$phaseInc <- relevel(aData$phaseInc, ref = "testA-B")

a5 <- lmer(logTime~ poly(TargetX,2) * poly(TargetY,2) + phaseSlide + phaseInc + (1|Participant) + (1|backgroundID), data=aData)

summary(a5)

# phaseInctestAB-B -3.172e-02 6.210e-02 5.773e+03 -0.511 0.6095

# phaseInctestAB-A 2.648e-01 6.207e-02 5.792e+03 4.266 2.02e-05 ***

# phaseInctestB-B -3.923e-02 6.209e-02 5.790e+03 -0.632 0.5275

# phaseInctestA-A 3.256e-01 6.109e-02 6.607e+03 5.330 1.02e-07 ***

# phaseInclearning 3.231e-01 4.522e-02 6.622e+03 7.144 1.00e-12 ***

# phaseIncsearchA 2.874e-01 4.925e-02 7.556e+03 5.836 5.57e-09 ***

# phaseIncsearchAB 2.202e-01 5.407e-02 5.502e+03 4.072 4.73e-05 ***

# phaseIncsearchB -5.375e-03 5.405e-02 5.520e+03 -0.099 0.9208

# phaseInctestB-A 3.119e-01 6.213e-02 5.798e+03 5.021 5.30e-07 ***

# Relevel for testB-A

aData$phaseInc <- relevel(aData$phaseInc, ref = "testB-A")

a5 <- lmer(logTime~ poly(TargetX,2) * poly(TargetY,2) + phaseSlide + phaseInc + (1|Participant) + (1|backgroundID), data=aData)

summary(a5)

# phaseInctestA-B -3.119e-01 6.213e-02 5.798e+03 -5.021 5.30e-07 ***

# phaseInctestAB-B -3.437e-01 6.209e-02 5.804e+03 -5.535 3.25e-08 ***

# phaseInctestAB-A -4.716e-02 6.201e-02 5.807e+03 -0.760 0.4470

# phaseInctestB-B -3.512e-01 6.107e-02 6.631e+03 -5.750 9.33e-09 ***

# phaseInctestA-A 1.367e-02 6.209e-02 5.779e+03 0.220 0.8258

# phaseInclearning 1.112e-02 4.520e-02 6.645e+03 0.246 0.8057

# phaseIncsearchA -2.452e-02 5.403e-02 5.514e+03 -0.454 0.6500

# phaseIncsearchAB -9.178e-02 5.406e-02 5.528e+03 -1.698 0.0896 .

# phaseIncsearchB -3.173e-01 4.924e-02 7.560e+03 -6.444 1.23e-10 ***

# Relevel for learning

aData$phaseInc <- relevel(aData$phaseInc, ref = "learning")

a5 <- lmer(logTime~ poly(TargetX,2) * poly(TargetY,2) + phaseSlide + phaseInc + (1|Participant) + (1|backgroundID), data=aData)

summary(a5)

# significantly different from searchB & searchAB

# phaseInctestB-A -1.112e-02 4.520e-02 6.645e+03 -0.246 0.80571

# phaseInctestA-B -3.231e-01 4.522e-02 6.622e+03 -7.144 1.00e-12 ***

# phaseInctestAB-B -3.548e-01 4.523e-02 6.628e+03 -7.845 4.88e-15 ***

# phaseInctestAB-A -5.827e-02 4.517e-02 6.646e+03 -1.290 0.19704

# phaseInctestB-B -3.623e-01 4.522e-02 6.636e+03 -8.011 1.33e-15 ***

# phaseInctestA-A 2.547e-03 4.521e-02 6.601e+03 0.056 0.95507

# phaseIncsearchA -3.564e-02 3.335e-02 6.621e+03 -1.069 0.28526

# phaseIncsearchAB -1.029e-01 3.333e-02 6.632e+03 -3.087 0.00203 **

# phaseIncsearchB -3.284e-01 3.332e-02 6.644e+03 -9.856 < 2e-16 ***

# Relevel for searchA

aData$phaseInc <- relevel(aData$phaseInc, ref = "searchA")

a5 <- lmer(logTime~ poly(TargetX,2) * poly(TargetY,2) + phaseSlide + phaseInc + (1|Participant) + (1|backgroundID), data=aData)

summary(a5)

# significantly different from searchB

# phaseInclearning 3.564e-02 3.335e-02 6.621e+03 1.069 0.2853

# phaseInctestB-A 2.452e-02 5.403e-02 5.514e+03 0.454 0.6500

# phaseInctestA-B -2.874e-01 4.925e-02 7.556e+03 -5.836 5.57e-09 ***

# phaseInctestAB-B -3.191e-01 5.406e-02 5.497e+03 -5.903 3.78e-09 ***

# phaseInctestAB-A -2.264e-02 5.402e-02 5.523e+03 -0.419 0.6752

# phaseInctestB-B -3.267e-01 5.405e-02 5.513e+03 -6.044 1.60e-09 ***

# phaseInctestA-A 3.818e-02 4.924e-02 7.557e+03 0.775 0.4381

# phaseIncsearchAB -6.726e-02 4.460e-02 4.976e+03 -1.508 0.1316

# phaseIncsearchB -2.928e-01 4.459e-02 4.987e+03 -6.566 5.68e-11 ***

# ---------------Plotting Exp2A Results--------------------

# order for graphing:

aData$phaseInc <- relevel(aData$phaseInc, ref = "testAB-B")

aData$phaseInc <- relevel(aData$phaseInc, ref = "testB-B")

aData$phaseInc <- relevel(aData$phaseInc, ref = "testA-B")

aData$phaseInc <- relevel(aData$phaseInc, ref = "testAB-A")

aData$phaseInc <- relevel(aData$phaseInc, ref = "testB-A")

aData$phaseInc <- relevel(aData$phaseInc, ref = "testA-A")

aData$phaseInc <- relevel(aData$phaseInc, ref = "searchAB")

aData$phaseInc <- relevel(aData$phaseInc, ref = "searchB")

aData$phaseInc <- relevel(aData$phaseInc, ref = "searchA")

aData$phaseInc <- relevel(aData$phaseInc, ref = "learning")

ggplot(aData, aes(phaseInc, timeTo20, fill=trialPhase)) +

geom_boxplot() +

labs(y="Capture Time (ms)")+

labs(x="Trial Phase")+

scale_y_continuous(trans = "log", breaks = c(800,1600,3200,6400,12800)) +

theme(panel.grid.major = element_blank(), panel.grid.minor = element_blank(), panel.background = element_blank(), panel.border = element_rect(colour = "black", fill=NA) )

# This graph is way easier to interpret, ending the test with A or B explains the pattern completely, i.e. there's no evidence for

# a difference in search image (prior experience), likewise there's no evidence for learning in disruptive at all.

#--------------------EXP2B DISTRACTIVE VS BACKGROUND MATCHING-----------------------

# "A" = Distractive

# "B" = Background Matching

b1 <- lmer(logTime~ poly(TargetX,2) * poly(TargetY,2) + inverted + phaseSlide * phaseInc + (1|Participant) + (1|backgroundID), data=bData)

summary(b1)

pamer.fnc(b1)

b2 <- lmer(logTime~ poly(TargetX,2) * poly(TargetY,2) + inverted + phaseSlide + phaseInc + (1|Participant) + (1|backgroundID), data=bData)

summary(b2)

pamer.fnc(b2)

anova(b1,b2) # There is evidecne for a learning effect model comparison Chi square = 24.004 df = 9, p = 0.004295

b3 <- lmer(logTime~ poly(TargetX,2) * poly(TargetY,2) + phaseSlide * phaseInc + (1|Participant) + (1|backgroundID), data=bData)

summary(b3)

pamer.fnc(b3)

anova(b3,b1) # NS, leave inversion out Chi Sqaure = 1.1685, df = 1, p = 0.2797

# ----------------------EXP2B final distractive vs BM model--------------------------

#Make planned comparisons between test treatments with moth treatment removed (incase it interacts with phaseInc)

# Relevel for testA-A

bData$phaseInc <- relevel(bData$phaseInc, ref = "testA-A")

b3 <- lmer(logTime~ poly(TargetX,2) * poly(TargetY,2) + phaseSlide * phaseInc + (1|Participant) + (1|backgroundID), data=bData)

summary(b3)

# phaseInclearning 3.182e-01 6.849e-02 7.413e+03 4.646 3.43e-06 ***

# phaseIncsearchA -1.327e-02 7.970e-02 7.357e+03 -0.166 0.867817

# phaseIncsearchB 1.405e-01 8.163e-02 7.404e+03 1.722 0.085176 .

# phaseIncsearchAB 6.455e-02 8.194e-02 7.406e+03 0.788 0.430835

# phaseInctestB-A 1.300e-01 9.396e-02 7.412e+03 1.384 0.166383

# phaseInctestAB-A 8.622e-02 9.505e-02 7.408e+03 0.907 0.364424

# phaseInctestA-B 2.606e-01 9.460e-02 7.410e+03 2.755 0.005883 **

# phaseInctestB-B 3.368e-01 9.423e-02 7.408e+03 3.574 0.000353 ***

# phaseInctestAB-B 1.024e-01 9.395e-02 7.413e+03 1.090 0.275946

# phaseSlide:phaseInclearning -8.514e-03 6.693e-03 7.297e+03 -1.272 0.203414

# phaseSlide:phaseIncsearchA 5.186e-03 8.152e-03 7.299e+03 0.636 0.524646

# phaseSlide:phaseIncsearchB 3.061e-03 8.133e-03 7.299e+03 0.376 0.706638

# phaseSlide:phaseIncsearchAB 5.601e-03 8.159e-03 7.300e+03 0.686 0.492432

# phaseSlide:phaseInctestB-A -8.670e-03 9.370e-03 7.298e+03 -0.925 0.354802

# phaseSlide:phaseInctestAB-A -7.263e-03 9.485e-03 7.295e+03 -0.766 0.443879

# phaseSlide:phaseInctestA-B -2.067e-03 9.490e-03 7.297e+03 -0.218 0.827592

# phaseSlide:phaseInctestB-B -1.147e-02 9.388e-03 7.296e+03 -1.222 0.221762

# phaseSlide:phaseInctestAB-B 3.381e-04 9.377e-03 7.300e+03 0.036 0.971241

# No learning effects

# Relevel for testB-B

bData$phaseInc <- relevel(bData$phaseInc, ref = "testB-B")

b3 <- lmer(logTime~ poly(TargetX,2) * poly(TargetY,2) + phaseSlide * phaseInc + (1|Participant) + (1|backgroundID), data=bData)

summary(b3)

# phaseInctestA-A -3.368e-01 9.423e-02 7.408e+03 -3.574 0.000353 ***

# phaseInclearning -1.859e-02 6.895e-02 7.410e+03 -0.270 0.787423

# phaseIncsearchA -3.501e-01 8.226e-02 7.403e+03 -4.256 2.11e-05 ***

# phaseIncsearchB -1.963e-01 7.984e-02 7.352e+03 -2.459 0.013967 *

# phaseIncsearchAB -2.723e-01 8.233e-02 7.405e+03 -3.307 0.000947 ***

# phaseInctestB-A -2.068e-01 9.382e-02 7.411e+03 -2.204 0.027559 *

# phaseInctestAB-A -2.506e-01 9.544e-02 7.408e+03 -2.626 0.008661 **

# phaseInctestA-B -7.621e-02 9.552e-02 7.411e+03 -0.798 0.424962

# phaseInctestAB-B -2.345e-01 9.422e-02 7.408e+03 -2.489 0.012847 *

# phaseSlide:phaseInctestA-A 1.147e-02 9.388e-03 7.296e+03 1.222 0.221762

# phaseSlide:phaseInclearning 2.959e-03 6.731e-03 7.298e+03 0.440 0.660275

# phaseSlide:phaseIncsearchA 1.666e-02 8.178e-03 7.298e+03 2.037 0.041682 *

# phaseSlide:phaseIncsearchB 1.453e-02 8.158e-03 7.297e+03 1.782 0.074860 .

# phaseSlide:phaseIncsearchAB 1.707e-02 8.186e-03 7.300e+03 2.086 0.037051 *

# phaseSlide:phaseInctestB-A 2.802e-03 9.398e-03 7.299e+03 0.298 0.765610

# phaseSlide:phaseInctestAB-A 4.210e-03 9.516e-03 7.297e+03 0.442 0.658240

# phaseSlide:phaseInctestA-B 9.405e-03 9.522e-03 7.300e+03 0.988 0.323301

# phaseSlide:phaseInctestAB-B 1.181e-02 9.393e-03 7.298e+03 1.257 0.208670

# Relevel for testAB-A

bData$phaseInc <- relevel(bData$phaseInc, ref = "testAB-A")

b3 <- lmer(logTime~ poly(TargetX,2) * poly(TargetY,2) + phaseSlide * phaseInc + (1|Participant) + (1|backgroundID), data=bData)

summary(b3)

# phaseInctestB-B 2.506e-01 9.544e-02 7.408e+03 2.626 0.008661 **

# phaseInctestA-A -8.622e-02 9.505e-02 7.408e+03 -0.907 0.364424

# phaseInclearning 2.320e-01 7.010e-02 7.410e+03 3.310 0.000939 ***

# phaseIncsearchA -9.948e-02 8.322e-02 7.403e+03 -1.195 0.231954

# phaseIncsearchB 5.432e-02 8.301e-02 7.405e+03 0.654 0.512909

# phaseIncsearchAB -2.166e-02 8.117e-02 7.357e+03 -0.267 0.789573

# phaseInctestB-A 4.383e-02 9.509e-02 7.410e+03 0.461 0.644879

# phaseInctestA-B 1.744e-01 9.633e-02 7.411e+03 1.811 0.070257 .

# phaseInctestAB-B 1.614e-02 9.467e-02 7.412e+03 0.171 0.864613

# phaseSlide:phaseInctestB-B -4.210e-03 9.516e-03 7.297e+03 -0.442 0.658240

# phaseSlide:phaseInctestA-A 7.263e-03 9.485e-03 7.295e+03 0.766 0.443879

# phaseSlide:phaseInclearning -1.251e-03 6.866e-03 7.297e+03 -0.182 0.855432

# phaseSlide:phaseIncsearchA 1.245e-02 8.289e-03 7.297e+03 1.502 0.133182

# phaseSlide:phaseIncsearchB 1.032e-02 8.274e-03 7.298e+03 1.248 0.212156

# phaseSlide:phaseIncsearchAB 1.286e-02 8.304e-03 7.301e+03 1.549 0.121423

# phaseSlide:phaseInctestB-A -1.408e-03 9.490e-03 7.297e+03 -0.148 0.882084

# phaseSlide:phaseInctestA-B 5.196e-03 9.612e-03 7.297e+03 0.541 0.588826

# phaseSlide:phaseInctestAB-B 7.601e-03 9.507e-03 7.302e+03 0.800 0.424006

# Relevel for testAB-B

bData$phaseInc <- relevel(bData$phaseInc, ref = "testAB-B")

b3 <- lmer(logTime~ poly(TargetX,2) * poly(TargetY,2) + phaseSlide * phaseInc + (1|Participant) + (1|backgroundID), data=bData)

summary(b3)

# phaseInctestAB-A -1.614e-02 9.467e-02 7.412e+03 -0.171 0.86461

# phaseInctestB-B 2.345e-01 9.422e-02 7.408e+03 2.489 0.01285 *

# phaseInctestA-A -1.024e-01 9.395e-02 7.413e+03 -1.090 0.27595

# phaseInclearning 2.159e-01 6.852e-02 7.414e+03 3.151 0.00164 **

# phaseIncsearchA -1.156e-01 8.191e-02 7.407e+03 -1.412 0.15809

# phaseIncsearchB 3.817e-02 8.163e-02 7.405e+03 0.468 0.64005

# phaseIncsearchAB -3.781e-02 7.979e-02 7.357e+03 -0.474 0.63564

# phaseInctestB-A 2.769e-02 9.401e-02 7.414e+03 0.295 0.76837

# phaseInctestA-B 1.583e-01 9.521e-02 7.414e+03 1.662 0.09653 .

# phaseSlide:phaseInctestAB-A -7.601e-03 9.506e-03 7.302e+03 -0.800 0.42401

# phaseSlide:phaseInctestB-B -1.181e-02 9.393e-03 7.298e+03 -1.257 0.20867

# phaseSlide:phaseInctestA-A -3.381e-04 9.377e-03 7.300e+03 -0.036 0.97124

# phaseSlide:phaseInclearning -8.852e-03 6.706e-03 7.302e+03 -1.320 0.18687

# phaseSlide:phaseIncsearchA 4.848e-03 8.161e-03 7.301e+03 0.594 0.55248

# phaseSlide:phaseIncsearchB 2.723e-03 8.137e-03 7.300e+03 0.335 0.73791

# phaseSlide:phaseIncsearchAB 5.263e-03 8.172e-03 7.305e+03 0.644 0.51958

# phaseSlide:phaseInctestB-A -9.008e-03 9.379e-03 7.300e+03 -0.960 0.33684

# phaseSlide:phaseInctestA-B -2.405e-03 9.503e-03 7.302e+03 -0.253 0.80022

# Relevel for testA-B

bData$phaseInc <- relevel(bData$phaseInc, ref = "testA-B")

b3 <- lmer(logTime~ poly(TargetX,2) * poly(TargetY,2) + phaseSlide * phaseInc + (1|Participant) + (1|backgroundID), data=bData)

summary(b3)

# phaseInctestAB-B -1.583e-01 9.521e-02 7.414e+03 -1.662 0.096529 .

# phaseInctestAB-A -1.744e-01 9.633e-02 7.411e+03 -1.811 0.070257 .

# phaseInctestB-B 7.621e-02 9.552e-02 7.411e+03 0.798 0.424962

# phaseInctestA-A -2.606e-01 9.460e-02 7.410e+03 -2.755 0.005883 **

# phaseInclearning 5.762e-02 7.020e-02 7.415e+03 0.821 0.411810

# phaseIncsearchA -2.739e-01 8.108e-02 7.358e+03 -3.378 0.000734 ***

# phaseIncsearchB -1.201e-01 8.312e-02 7.408e+03 -1.445 0.148573

# phaseIncsearchAB -1.961e-01 8.332e-02 7.405e+03 -2.353 0.018644 *

# phaseInctestB-A -1.306e-01 9.518e-02 7.413e+03 -1.372 0.170151

# phaseSlide:phaseInctestAB-B 2.405e-03 9.503e-03 7.302e+03 0.253 0.800218

# phaseSlide:phaseInctestAB-A -5.196e-03 9.612e-03 7.297e+03 -0.541 0.588826

# phaseSlide:phaseInctestB-B -9.405e-03 9.522e-03 7.300e+03 -0.988 0.323301

# phaseSlide:phaseInctestA-A 2.067e-03 9.490e-03 7.297e+03 0.218 0.827592

# phaseSlide:phaseInclearning -6.447e-03 6.871e-03 7.301e+03 -0.938 0.348162

# phaseSlide:phaseIncsearchA 7.253e-03 8.296e-03 7.301e+03 0.874 0.381982

# phaseSlide:phaseIncsearchB 5.128e-03 8.286e-03 7.303e+03 0.619 0.536002

# phaseSlide:phaseIncsearchAB 7.668e-03 8.302e-03 7.300e+03 0.924 0.355700

# phaseSlide:phaseInctestB-A -6.604e-03 9.498e-03 7.300e+03 -0.695 0.486937

# Relevel for testB-A

bData$phaseInc <- relevel(bData$phaseInc, ref = "testB-A")

b3 <- lmer(logTime~ poly(TargetX,2) * poly(TargetY,2) + phaseSlide * phaseInc + (1|Participant) + (1|backgroundID), data=bData)

summary(b3)

# phaseInctestA-B 1.306e-01 9.518e-02 7.413e+03 1.372 0.17015

# phaseInctestAB-B -2.769e-02 9.401e-02 7.414e+03 -0.295 0.76837

# phaseInctestAB-A -4.383e-02 9.509e-02 7.410e+03 -0.461 0.64488

# phaseInctestB-B 2.068e-01 9.382e-02 7.411e+03 2.204 0.02756 *

# phaseInctestA-A -1.300e-01 9.396e-02 7.412e+03 -1.384 0.16638

# phaseInclearning 1.882e-01 6.850e-02 7.412e+03 2.747 0.00602 **

# phaseIncsearchA -1.433e-01 8.195e-02 7.407e+03 -1.749 0.08037 .

# phaseIncsearchB 1.049e-02 7.954e-02 7.357e+03 0.132 0.89511

# phaseIncsearchAB -6.549e-02 8.204e-02 7.409e+03 -0.798 0.42471

# phaseSlide:phaseInctestA-B 6.603e-03 9.498e-03 7.300e+03 0.695 0.48694

# phaseSlide:phaseInctestAB-B 9.008e-03 9.379e-03 7.300e+03 0.960 0.33684

# phaseSlide:phaseInctestAB-A 1.408e-03 9.490e-03 7.297e+03 0.148 0.88208

# phaseSlide:phaseInctestB-B -2.802e-03 9.398e-03 7.299e+03 -0.298 0.76561

# phaseSlide:phaseInctestA-A 8.670e-03 9.370e-03 7.298e+03 0.925 0.35480

# phaseSlide:phaseInclearning 1.568e-04 6.698e-03 7.299e+03 0.023 0.98133

# phaseSlide:phaseIncsearchA 1.386e-02 8.159e-03 7.301e+03 1.698 0.08948 .

# phaseSlide:phaseIncsearchB 1.173e-02 8.140e-03 7.300e+03 1.441 0.14954

# Relevel for learning

bData$phaseInc <- relevel(bData$phaseInc, ref = "learning")

b3 <- lmer(logTime~ poly(TargetX,2) * poly(TargetY,2) + phaseSlide * phaseInc + (1|Participant) + (1|backgroundID), data=bData)

summary(b3) # significantly different from searchB & searchAB

# phaseInctestB-A -1.882e-01 6.850e-02 7.412e+03 -2.747 0.006024 **

# phaseInctestA-B -5.762e-02 7.020e-02 7.415e+03 -0.821 0.411810

# phaseInctestAB-B -2.159e-01 6.852e-02 7.414e+03 -3.151 0.001636 **

# phaseInctestAB-A -2.320e-01 7.010e-02 7.410e+03 -3.310 0.000939 ***

# phaseInctestB-B 1.859e-02 6.895e-02 7.410e+03 0.270 0.787423

# phaseInctestA-A -3.182e-01 6.849e-02 7.413e+03 -4.646 3.43e-06 ***

# phaseIncsearchA -3.315e-01 5.076e-02 7.416e+03 -6.531 6.97e-11 ***

# phaseIncsearchB -1.777e-01 5.036e-02 7.414e+03 -3.529 0.000420 ***

# phaseIncsearchAB -2.537e-01 5.083e-02 7.415e+03 -4.990 6.17e-07 ***

# poly(TargetX, 2)1:poly(TargetY, 2)1 6.026e+01 4.757e+01 7.340e+03 1.267 0.205233

# phaseSlide:phaseInctestB-A -1.568e-04 6.698e-03 7.299e+03 -0.023 0.981330

# phaseSlide:phaseInctestA-B 6.447e-03 6.871e-03 7.301e+03 0.938 0.348162

# phaseSlide:phaseInctestAB-B 8.852e-03 6.706e-03 7.302e+03 1.320 0.186871

# phaseSlide:phaseInctestAB-A 1.251e-03 6.866e-03 7.297e+03 0.182 0.855432

# phaseSlide:phaseInctestB-B -2.959e-03 6.731e-03 7.298e+03 -0.440 0.660275

# phaseSlide:phaseInctestA-A 8.514e-03 6.693e-03 7.297e+03 1.272 0.203414

# phaseSlide:phaseIncsearchA 1.370e-02 4.848e-03 7.300e+03 2.826 0.004729 **

# phaseSlide:phaseIncsearchB 1.157e-02 4.819e-03 7.301e+03 2.402 0.016332 *

# phaseSlide:phaseIncsearchAB 1.411e-02 4.860e-03 7.302e+03 2.904 0.003690 **

# Relevel for searchA

bData$phaseInc <- relevel(bData$phaseInc, ref = "searchA")

b3 <- lmer(logTime~ poly(TargetX,2) * poly(TargetY,2) + phaseSlide * phaseInc + (1|Participant) + (1|backgroundID), data=bData)

summary(b3) # significantly different from searchB & searchAB

# phaseInclearning 3.315e-01 5.076e-02 7.416e+03 6.531 6.97e-11 ***

# phaseInctestB-A 1.433e-01 8.195e-02 7.407e+03 1.749 0.080369 .

# phaseInctestA-B 2.739e-01 8.108e-02 7.358e+03 3.378 0.000734 ***

# phaseInctestAB-B 1.156e-01 8.191e-02 7.407e+03 1.412 0.158086

# phaseInctestAB-A 9.948e-02 8.322e-02 7.403e+03 1.195 0.231954

# phaseInctestB-B 3.501e-01 8.226e-02 7.403e+03 4.256 2.11e-05 ***

# phaseInctestA-A 1.327e-02 7.970e-02 7.357e+03 0.166 0.867817

# phaseIncsearchB 1.538e-01 6.745e-02 7.384e+03 2.280 0.022629 *

# phaseIncsearchAB 7.782e-02 6.785e-02 7.390e+03 1.147 0.251484

# phaseSlide:phaseInclearning -1.370e-02 4.848e-03 7.300e+03 -2.826 0.004729 **

# phaseSlide:phaseInctestB-A -1.386e-02 8.159e-03 7.301e+03 -1.698 0.089482 .

# phaseSlide:phaseInctestA-B -7.253e-03 8.296e-03 7.301e+03 -0.874 0.381982

# phaseSlide:phaseInctestAB-B -4.848e-03 8.161e-03 7.301e+03 -0.594 0.552483

# phaseSlide:phaseInctestAB-A -1.245e-02 8.289e-03 7.297e+03 -1.502 0.133182

# phaseSlide:phaseInctestB-B -1.666e-02 8.178e-03 7.298e+03 -2.037 0.041682 *

# phaseSlide:phaseInctestA-A -5.186e-03 8.152e-03 7.299e+03 -0.636 0.524646

# phaseSlide:phaseIncsearchB -2.125e-03 6.694e-03 7.299e+03 -0.317 0.750890

# phaseSlide:phaseIncsearchAB 4.146e-04 6.731e-03 7.304e+03 0.062 0.950891

# Relevel for searchAB

bData$phaseInc <- relevel(bData$phaseInc, ref = "searchAB")

b3 <- lmer(logTime~ poly(TargetX,2) * poly(TargetY,2) + phaseSlide * phaseInc + (1|Participant) + (1|backgroundID), data=bData)

summary(b3)

# phaseIncsearchA -7.782e-02 6.785e-02 7.390e+03 -1.147 0.251484

# phaseInclearning 2.537e-01 5.083e-02 7.415e+03 4.990 6.17e-07 ***

# phaseInctestB-A 6.549e-02 8.204e-02 7.409e+03 0.798 0.424707

# phaseInctestA-B 1.961e-01 8.332e-02 7.405e+03 2.353 0.018644 *

# phaseInctestAB-B 3.781e-02 7.979e-02 7.357e+03 0.474 0.635644

# phaseInctestAB-A 2.166e-02 8.117e-02 7.357e+03 0.267 0.789573

# phaseInctestB-B 2.723e-01 8.233e-02 7.405e+03 3.307 0.000947 ***

# phaseInctestA-A -6.455e-02 8.194e-02 7.406e+03 -0.788 0.430835

# phaseIncsearchB 7.598e-02 6.757e-02 7.391e+03 1.124 0.260872

# phaseSlide:phaseIncsearchA -4.146e-04 6.731e-03 7.304e+03 -0.062 0.950891

# phaseSlide:phaseInclearning -1.411e-02 4.860e-03 7.302e+03 -2.904 0.003690 **

# phaseSlide:phaseInctestB-A -1.427e-02 8.164e-03 7.301e+03 -1.748 0.080508 .

# phaseSlide:phaseInctestA-B -7.668e-03 8.301e-03 7.300e+03 -0.924 0.355700

# phaseSlide:phaseInctestAB-B -5.263e-03 8.172e-03 7.305e+03 -0.644 0.519578

# phaseSlide:phaseInctestAB-A -1.286e-02 8.304e-03 7.301e+03 -1.549 0.121423

# phaseSlide:phaseInctestB-B -1.707e-02 8.186e-03 7.300e+03 -2.086 0.037051 *

# phaseSlide:phaseInctestA-A -5.601e-03 8.159e-03 7.300e+03 -0.686 0.492432

# phaseSlide:phaseIncsearchB -2.540e-03 6.712e-03 7.305e+03 -0.378 0.705152

# ---------------Plotting Exp2B Results--------------------

# order for graphing:

bData$phaseInc <- relevel(bData$phaseInc, ref = "testAB-B")

bData$phaseInc <- relevel(bData$phaseInc, ref = "testB-B")

bData$phaseInc <- relevel(bData$phaseInc, ref = "testA-B")

bData$phaseInc <- relevel(bData$phaseInc, ref = "testAB-A")

bData$phaseInc <- relevel(bData$phaseInc, ref = "testB-A")

bData$phaseInc <- relevel(bData$phaseInc, ref = "testA-A")

bData$phaseInc <- relevel(bData$phaseInc, ref = "searchAB")

bData$phaseInc <- relevel(bData$phaseInc, ref = "searchB")

bData$phaseInc <- relevel(bData$phaseInc, ref = "searchA")

bData$phaseInc <- relevel(bData$phaseInc, ref = "learning")

ggplot(bData, aes(phaseInc, timeTo20, fill=trialPhase)) +

geom_boxplot() +

labs(y="Capture Time (ms)")+

labs(x="Trial Phase")+

scale_y_continuous(trans = "log", breaks = c(800,1600,3200,6400,12800)) +

theme(panel.grid.major = element_blank(), panel.grid.minor = element_blank(), panel.background = element_blank(), panel.border = element_rect(colour = "black", fill=NA) )

library(car)

coplot(logTime ~ phaseSlide | phaseInc, panel=panel.car, lowess.line=FALSE, col="red", data=bData)

require(gridExtra)

tempData <- subset(bData, phaseInc == "learning")

p1 <- ggplot(tempData, aes(x=phaseSlide, y=logTime)) + geom_point(shape=19, color="#990000", alpha=0.3) +

geom_smooth(method=lm, se=TRUE, fill="#990000", alpha=0.5, color="#990000") +

labs(y="Log Capture Time (ms)")+

labs(x="Learning Phase Slides")+

coord_cartesian(ylim = c(6, log(21000))) +

theme(panel.grid.major = element_blank(), panel.grid.minor = element_blank(), panel.background = element_blank(), panel.border = element_rect(colour = "black", fill=NA))

tempData <- subset(bData, phaseInc == "searchA")

p2 <- ggplot(tempData, aes(x=phaseSlide, y=logTime)) + geom_point(shape=19, color="#009900", alpha=0.3) +

geom_smooth(method=lm,se=TRUE, fill="#009900", alpha=0.5, color="#009900") +

labs(x="Dac")+

coord_cartesian(ylim = c(6, log(21000))) +

theme(panel.grid.major = element_blank(), panel.grid.minor = element_blank(), panel.background = element_blank(), panel.border = element_rect(colour = "black", fill=NA), axis.ticks.y = element_blank(), axis.text.y = element_blank(), axis.title.y = element_blank())

tempData <- subset(bData, phaseInc == "searchB")

p3 <- ggplot(tempData, aes(x=phaseSlide, y=logTime)) + geom_point(shape=19, color="#009900", alpha=0.3) +

geom_smooth(method=lm,se=TRUE, fill="#009900", alpha=0.5, color="#009900") +

labs(x="BM")+

coord_cartesian(ylim = c(6, log(21000))) +

theme(panel.grid.major = element_blank(), panel.grid.minor = element_blank(), panel.background = element_blank(), panel.border = element_rect(colour = "black", fill=NA), axis.ticks.y = element_blank(), axis.text.y = element_blank(), axis.title.y = element_blank())

tempData <- subset(bData, phaseInc == "searchAB")

p4 <- ggplot(tempData, aes(x=phaseSlide, y=logTime)) + geom_point(shape=19, color="#009900", alpha=0.3) +

geom_smooth(method=lm,se=TRUE, fill="#009900", alpha=0.5, color="#009900") +

labs(x="Dac/BM")+

coord_cartesian(ylim = c(6, log(21000))) +

theme(panel.grid.major = element_blank(), panel.grid.minor = element_blank(), panel.background = element_blank(), panel.border = element_rect(colour = "black", fill=NA), axis.ticks.y = element_blank(), axis.text.y = element_blank(), axis.title.y = element_blank())

pa <- grid.arrange(p1, p2, p3, p4, ncol=4, widths=c(2,1,1,1) )

tempData <- subset(bData, phaseInc == "testA-A")

p5 <- ggplot(tempData, aes(x=phaseSlide, y=logTime)) + geom_point(shape=19, color="#000099", alpha=0.3) +

geom_smooth(method=lm,se=TRUE, fill="#000099", alpha=0.5, color="#000099") +

labs(y="Log Capture Time (ms)")+

labs(x="Dac->Dac")+

coord_cartesian(ylim = c(6, log(21000))) +

theme(panel.grid.major = element_blank(), panel.grid.minor = element_blank(), panel.background = element_blank(), panel.border = element_rect(colour = "black", fill=NA))

tempData <- subset(bData, phaseInc == "testB-A")

p6 <- ggplot(tempData, aes(x=phaseSlide, y=logTime)) + geom_point(shape=19, color="#000099", alpha=0.3) +

geom_smooth(method=lm,se=TRUE, fill="#000099", alpha=0.5, color="#000099") +

labs(x="BM->Dac")+

coord_cartesian(ylim = c(6, log(21000))) +

theme(panel.grid.major = element_blank(), panel.grid.minor = element_blank(), panel.background = element_blank(), panel.border = element_rect(colour = "black", fill=NA), axis.ticks.y = element_blank(), axis.text.y = element_blank(), axis.title.y = element_blank())

tempData <- subset(bData, phaseInc == "testAB-A")

p7 <- ggplot(tempData, aes(x=phaseSlide, y=logTime)) + geom_point(shape=19, color="#000099", alpha=0.3) +

geom_smooth(method=lm,se=TRUE, fill="#000099", alpha=0.5, color="#000099") +

labs(x="Dac/BM->Dac")+

coord_cartesian(ylim = c(6, log(21000))) +

theme(panel.grid.major = element_blank(), panel.grid.minor = element_blank(), panel.background = element_blank(), panel.border = element_rect(colour = "black", fill=NA), axis.ticks.y = element_blank(), axis.text.y = element_blank(), axis.title.y = element_blank())

tempData <- subset(bData, phaseInc == "testA-B")

p8 <- ggplot(tempData, aes(x=phaseSlide, y=logTime)) + geom_point(shape=19, color="#000099", alpha=0.3) +

geom_smooth(method=lm,se=TRUE, fill="#000099", alpha=0.5, color="#000099") +

labs(x="Dac->BM")+

coord_cartesian(ylim = c(6, log(21000))) +

theme(panel.grid.major = element_blank(), panel.grid.minor = element_blank(), panel.background = element_blank(), panel.border = element_rect(colour = "black", fill=NA), axis.ticks.y = element_blank(), axis.text.y = element_blank(), axis.title.y = element_blank())

tempData <- subset(bData, phaseInc == "testB-B")

p9 <- ggplot(tempData, aes(x=phaseSlide, y=logTime)) + geom_point(shape=19, color="#000099", alpha=0.3) +

geom_smooth(method=lm,se=TRUE, fill="#000099", alpha=0.5, color="#000099") +

labs(x="BM->BM")+

coord_cartesian(ylim = c(6, log(21000))) +

theme(panel.grid.major = element_blank(), panel.grid.minor = element_blank(), panel.background = element_blank(), panel.border = element_rect(colour = "black", fill=NA), axis.ticks.y = element_blank(), axis.text.y = element_blank(), axis.title.y = element_blank())

tempData <- subset(bData, phaseInc == "testAB-B")

p10 <- ggplot(tempData, aes(x=phaseSlide, y=logTime)) + geom_point(shape=19, color="#000099", alpha=0.3) +

geom_smooth(method=lm,se=TRUE, fill="#000099", alpha=0.5, color="#000099") +

labs(x="Dac/BM->BM")+

coord_cartesian(ylim = c(6, log(21000))) +

theme(panel.grid.major = element_blank(), panel.grid.minor = element_blank(), panel.background = element_blank(), panel.border = element_rect(colour = "black", fill=NA), axis.ticks.y = element_blank(), axis.text.y = element_blank(), axis.title.y = element_blank())

pb <- grid.arrange(p5, p6, p7, p8, p9, p10, ncol=6, widths=c(1.15,1,1,1,1,1) )

grid.arrange(pa,pb, ncol=1)

#--------------------EXP2C DISRUPTIVE VS DISTRACTIVE-----------------------

# "A" = Disruptive

# "B" = Distractive

c1 <- lmer(logTime~ poly(TargetX,2) * poly(TargetY,2) + inverted + phaseSlide * phaseInc + (1|Participant) + (1|backgroundID), data=cData)

summary(c1)

pamer.fnc(c1)

mcp.fnc(c1)

c2 <- lmer(logTime~ poly(TargetX,2) * poly(TargetY,2) + inverted + phaseSlide + phaseInc + (1|Participant) + (1|backgroundID), data=cData)

summary(c2)

pamer.fnc(c2)

anova(c1,c2) # no diff, no learning effects Chi = 10.345, df = 9, p = 0.3233

c3 <- lmer(logTime~ poly(TargetX,2) * poly(TargetY,2) + phaseSlide + phaseInc + (1|Participant) + (1|backgroundID), data=cData)

summary(c3)

pamer.fnc(c3)

anova(c2,c3) # inverted is significant, but AIC & BIC disagree on which model is better

c4 <- lmer(logTime~ poly(TargetX,2) * poly(TargetY,2) + phaseSlide + inverted * phaseInc + (1|Participant) + (1|backgroundID), data=cData)

summary(c4)

pamer.fnc(c4)

anova(c2,c4) # better to leave out interaction

# ----------------------EXP2C final disruptive vs distractive model--------------------------

#Make planned comparisons between test treatments with moth treatment removed (incase it interacts with phaseInc)

# Relevel for testA-A

cData$phaseInc <- relevel(cData$phaseInc, ref = "testA-A")

c3 <- lmer(logTime~ poly(TargetX,2) * poly(TargetY,2) + phaseSlide + phaseInc + (1|Participant) + (1|backgroundID), data=cData)

summary(c3)

# phaseInclearning -5.356e-02 4.322e-02 7.244e+03 -1.239 0.2153

# phaseIncsearchA 2.772e-02 4.679e-02 7.613e+03 0.592 0.5536

# phaseIncsearchAB -2.245e-01 5.191e-02 6.605e+03 -4.325 1.54e-05 ***

# phaseIncsearchB -4.723e-01 5.192e-02 6.595e+03 -9.097 < 2e-16 ***

# phaseInctestA-B -4.079e-01 5.836e-02 7.275e+03 -6.990 3.00e-12 ***

# phaseInctestAB-A 7.250e-02 6.032e-02 6.805e+03 1.202 0.2294

# phaseInctestAB-B -4.989e-01 5.889e-02 6.775e+03 -8.473 < 2e-16 ***

# phaseInctestB-A 1.315e-01 5.958e-02 6.785e+03 2.207 0.0273 *

# phaseInctestB-B -4.689e-01 5.955e-02 6.786e+03 -7.874 4.00e-15 ***

# Relevel for testB-B

cData$phaseInc <- relevel(cData$phaseInc, ref = "testB-B")

c3 <- lmer(logTime~ poly(TargetX,2) * poly(TargetY,2) + phaseSlide + phaseInc + (1|Participant) + (1|backgroundID), data=cData)

summary(c3)

# phaseInclearning 4.154e-01 4.320e-02 7.237e+03 9.615 < 2e-16 ***

# phaseIncsearchA 4.966e-01 5.193e-02 6.595e+03 9.563 < 2e-16 ***

# phaseIncsearchB -3.361e-03 4.675e-02 7.608e+03 -0.072 0.943

# phaseIncsearchAB 2.444e-01 5.192e-02 6.595e+03 4.707 2.57e-06 ***

# phaseInctestA-A 4.689e-01 5.955e-02 6.786e+03 7.874 4.00e-15 ***

# phaseInctestA-B 6.098e-02 5.952e-02 6.781e+03 1.024 0.306

# phaseInctestB-A 6.004e-01 5.836e-02 7.275e+03 10.289 < 2e-16 ***

# phaseInctestAB-A 5.414e-01 6.032e-02 6.797e+03 8.976 < 2e-16 ***

# phaseInctestAB-B -3.003e-02 5.885e-02 6.763e+03 -0.510 0.610

# Relevel for testAB-A

cData$phaseInc <- relevel(cData$phaseInc, ref = "testAB-A")

c3 <- lmer(logTime~ poly(TargetX,2) * poly(TargetY,2) + phaseSlide + phaseInc + (1|Participant) + (1|backgroundID), data=cData)

summary(c3)

# phaseInctestB-B -5.414e-01 6.032e-02 6.797e+03 -8.976 < 2e-16 ***

# phaseInclearning -1.261e-01 4.424e-02 7.237e+03 -2.849 0.00439 **

# phaseIncsearchA -4.478e-02 5.280e-02 6.621e+03 -0.848 0.39633

# phaseIncsearchB -5.448e-01 5.274e-02 6.615e+03 -10.330 < 2e-16 ***

# phaseIncsearchAB -2.970e-01 4.770e-02 7.608e+03 -6.228 4.99e-10 ***

# phaseInctestA-A -7.250e-02 6.032e-02 6.805e+03 -1.202 0.22942

# phaseInctestA-B -4.804e-01 6.031e-02 6.792e+03 -7.966 1.78e-15 ***

# phaseInctestB-A 5.901e-02 6.033e-02 6.793e+03 0.978 0.32800

# phaseInctestAB-B -5.714e-01 5.846e-02 7.273e+03 -9.775 < 2e-16 ***

# Relevel for testAB-B

cData$phaseInc <- relevel(cData$phaseInc, ref = "testAB-B")

c3 <- lmer(logTime~ poly(TargetX,2) * poly(TargetY,2) + phaseSlide + phaseInc + (1|Participant) + (1|backgroundID), data=cData)

summary(c3)

# phaseInclearning 4.454e-01 4.224e-02 7.232e+03 10.543 < 2e-16 ***

# phaseIncsearchA 5.267e-01 5.113e-02 6.564e+03 10.301 < 2e-16 ***

# phaseIncsearchB 2.667e-02 5.113e-02 6.563e+03 0.522 0.602

# phaseIncsearchAB 2.744e-01 4.586e-02 7.609e+03 5.984 2.28e-09 ***

# phaseInctestA-A 4.989e-01 5.889e-02 6.775e+03 8.473 < 2e-16 ***

# phaseInctestB-A 6.305e-01 5.890e-02 6.764e+03 10.705 < 2e-16 ***

# phaseInctestAB-A 5.714e-01 5.846e-02 7.273e+03 9.775 < 2e-16 ***

# phaseInctestA-B 9.101e-02 5.886e-02 6.765e+03 1.546 0.122

# phaseInctestB-B 3.003e-02 5.885e-02 6.763e+03 0.510 0.610

# Relevel for testA-B

cData$phaseInc <- relevel(cData$phaseInc, ref = "testA-B")

c3 <- lmer(logTime~ poly(TargetX,2) * poly(TargetY,2) + phaseSlide + phaseInc + (1|Participant) + (1|backgroundID), data=cData)

summary(c3)

# phaseInctestAB-B -9.101e-02 5.886e-02 6.765e+03 -1.546 0.122130

# phaseInclearning 3.544e-01 4.319e-02 7.236e+03 8.204 2.22e-16 ***

# phaseIncsearchA 4.357e-01 4.675e-02 7.610e+03 9.318 < 2e-16 ***

# phaseIncsearchB -6.434e-02 5.192e-02 6.588e+03 -1.239 0.215329

# phaseIncsearchAB 1.834e-01 5.190e-02 6.591e+03 3.534 0.000412 ***

# phaseInctestA-A 4.079e-01 5.836e-02 7.275e+03 6.990 3.00e-12 ***

# phaseInctestB-A 5.395e-01 5.954e-02 6.774e+03 9.060 < 2e-16 ***

# phaseInctestAB-A 4.804e-01 6.031e-02 6.792e+03 7.966 1.78e-15 ***

# phaseInctestB-B -6.098e-02 5.952e-02 6.781e+03 -1.024 0.305655

# Relevel for testB-A

cData$phaseInc <- relevel(cData$phaseInc, ref = "testB-A")

c3 <- lmer(logTime~ poly(TargetX,2) * poly(TargetY,2) + phaseSlide + phaseInc + (1|Participant) + (1|backgroundID), data=cData)

summary(c3)

# phaseInctestA-B -5.395e-01 5.954e-02 6.774e+03 -9.060 < 2e-16 ***

# phaseInctestAB-B -6.305e-01 5.890e-02 6.764e+03 -10.705 < 2e-16 ***

# phaseInclearning -1.851e-01 4.320e-02 7.230e+03 -4.284 1.86e-05 ***

# phaseIncsearchA -1.038e-01 5.194e-02 6.585e+03 -1.998 0.0457 *

# phaseIncsearchB -6.038e-01 4.677e-02 7.608e+03 -12.911 < 2e-16 ***

# phaseIncsearchAB -3.560e-01 5.194e-02 6.590e+03 -6.855 7.77e-12 ***

# phaseInctestA-A -1.315e-01 5.958e-02 6.785e+03 -2.207 0.0273 *

# phaseInctestAB-A -5.901e-02 6.033e-02 6.793e+03 -0.978 0.3280

# phaseInctestB-B -6.004e-01 5.836e-02 7.275e+03 -10.289 < 2e-16 ***

# Relevel for learning

cData$phaseInc <- relevel(cData$phaseInc, ref = "learning")

c3 <- lmer(logTime~ poly(TargetX,2) * poly(TargetY,2) + phaseSlide + phaseInc + (1|Participant) + (1|backgroundID), data=cData)

summary(c3)

# phaseInctestB-A 1.851e-01 4.320e-02 7.230e+03 4.284 1.86e-05 ***

# phaseInctestA-B -3.544e-01 4.319e-02 7.236e+03 -8.204 2.22e-16 ***

# phaseInctestAB-B -4.454e-01 4.224e-02 7.232e+03 -10.543 < 2e-16 ***

# phaseIncsearchA 8.128e-02 3.186e-02 7.199e+03 2.551 0.01075 *

# phaseIncsearchB -4.187e-01 3.188e-02 7.195e+03 -13.135 < 2e-16 ***

# phaseIncsearchAB -1.710e-01 3.184e-02 7.199e+03 -5.369 8.16e-08 ***

# phaseInctestA-A 5.356e-02 4.322e-02 7.244e+03 1.239 0.21529

# phaseInctestAB-A 1.261e-01 4.424e-02 7.237e+03 2.849 0.00439 **

# phaseInctestB-B -4.154e-01 4.320e-02 7.237e+03 -9.615 < 2e-16 ***

# Relevel for searchA

cData$phaseInc <- relevel(cData$phaseInc, ref = "searchA")

c3 <- lmer(logTime~ poly(TargetX,2) * poly(TargetY,2) + phaseSlide + phaseInc + (1|Participant) + (1|backgroundID), data=cData)

summary(c3)

# phaseInclearning -8.128e-02 3.186e-02 7.199e+03 -2.551 0.0108 *

# phaseInctestB-A 1.038e-01 5.194e-02 6.585e+03 1.998 0.0457 *

# phaseInctestA-B -4.357e-01 4.675e-02 7.610e+03 -9.318 < 2e-16 ***

# phaseInctestAB-B -5.267e-01 5.113e-02 6.564e+03 -10.301 < 2e-16 ***

# phaseIncsearchB -5.000e-01 4.298e-02 6.199e+03 -11.633 < 2e-16 ***

# phaseIncsearchAB -2.522e-01 4.295e-02 6.210e+03 -5.873 4.49e-09 ***

# phaseInctestA-A -2.772e-02 4.679e-02 7.613e+03 -0.592 0.5536

# phaseInctestAB-A 4.478e-02 5.280e-02 6.621e+03 0.848 0.3963

# phaseInctestB-B -4.966e-01 5.193e-02 6.595e+03 -9.563 < 2e-16 ***

# ---------------Plotting Exp2C Results--------------------

# order for graphing:

cData$phaseInc <- relevel(cData$phaseInc, ref = "testAB-B")

cData$phaseInc <- relevel(cData$phaseInc, ref = "testB-B")

cData$phaseInc <- relevel(cData$phaseInc, ref = "testA-B")

cData$phaseInc <- relevel(cData$phaseInc, ref = "testAB-A")

cData$phaseInc <- relevel(cData$phaseInc, ref = "testB-A")

cData$phaseInc <- relevel(cData$phaseInc, ref = "testA-A")

cData$phaseInc <- relevel(cData$phaseInc, ref = "searchAB")

cData$phaseInc <- relevel(cData$phaseInc, ref = "searchB")

cData$phaseInc <- relevel(cData$phaseInc, ref = "searchA")

cData$phaseInc <- relevel(cData$phaseInc, ref = "learning")

ggplot(cData, aes(phaseInc, timeTo20, fill=trialPhase)) +

geom_boxplot() +

labs(y="Capture Time (ms)")+

labs(x="Trial Phase")+

scale_y_continuous(trans = "log", breaks = c(800,1600,3200,6400,12800)) +

theme(panel.grid.major = element_blank(), panel.grid.minor = element_blank(), panel.background = element_blank(), panel.border = element_rect(colour = "black", fill=NA) )

#---------------------------Composite Exp2ABC boxplot--------------------------------

#Exp2A Results

# order for graphing:

aData$phaseInc <- relevel(aData$phaseInc, ref = "testAB-B")

aData$phaseInc <- relevel(aData$phaseInc, ref = "testB-B")

aData$phaseInc <- relevel(aData$phaseInc, ref = "testA-B")

aData$phaseInc <- relevel(aData$phaseInc, ref = "testAB-A")

aData$phaseInc <- relevel(aData$phaseInc, ref = "testB-A")

aData$phaseInc <- relevel(aData$phaseInc, ref = "testA-A")

aData$phaseInc <- relevel(aData$phaseInc, ref = "searchAB")

aData$phaseInc <- relevel(aData$phaseInc, ref = "searchB")

aData$phaseInc <- relevel(aData$phaseInc, ref = "searchA")

aData$phaseInc <- relevel(aData$phaseInc, ref = "learning")

bpa<-ggplot(aData, aes(phaseInc, timeTo20, fill=trialPhase)) +

geom_boxplot() +

labs(title="Background matching vs Disruptive")+

scale_x_discrete(labels=c("Learning", "Drp", "BM","Drp/BM","Drp->Drp","BM->Drp","Drp/BM->Drp","Drp->BM","BM->BM","Drp/BM->BM")) +

labs(y=" ")+

#labs(x="Trial Phase")+

scale_y_continuous(trans = "log", breaks = c(800,1600,3200,6400,12800)) +

theme(panel.grid.major = element_blank(), panel.grid.minor = element_blank(), panel.background = element_blank(), panel.border = element_rect(colour = "black", fill=NA), axis.title.x = element_blank(), axis.text.x=element_text(angle=-45, hjust = 0) )

#Exp2B Results

# order for graphing:

bData$phaseInc <- relevel(bData$phaseInc, ref = "testAB-B")

bData$phaseInc <- relevel(bData$phaseInc, ref = "testB-B")

bData$phaseInc <- relevel(bData$phaseInc, ref = "testA-B")

bData$phaseInc <- relevel(bData$phaseInc, ref = "testAB-A")

bData$phaseInc <- relevel(bData$phaseInc, ref = "testB-A")

bData$phaseInc <- relevel(bData$phaseInc, ref = "testA-A")

bData$phaseInc <- relevel(bData$phaseInc, ref = "searchAB")

bData$phaseInc <- relevel(bData$phaseInc, ref = "searchB")

bData$phaseInc <- relevel(bData$phaseInc, ref = "searchA")

bData$phaseInc <- relevel(bData$phaseInc, ref = "learning")

#A= Dac, B=BM

bpb<-ggplot(bData, aes(phaseInc, timeTo20, fill=trialPhase)) +

geom_boxplot() +

labs(title="Background matching vs Distractive")+

scale_x_discrete(labels=c("Learning", "Dac", "BM","Dac/BM","Dac->Dac","BM->Dac","Dac/BM->Dac","Dac->BM","BM->BM","Dac/BM->BM")) +

labs(y="Capture Time (ms)")+

#labs(x="Trial Phase")+

scale_y_continuous(trans = "log", breaks = c(800,1600,3200,6400,12800)) +

theme(panel.grid.major = element_blank(), panel.grid.minor = element_blank(), panel.background = element_blank(), panel.border = element_rect(colour = "black", fill=NA), axis.title.x = element_blank(), axis.text.x=element_text(angle=-45, hjust = 0) )

#Exp2C results

# order for graphing:

cData$phaseInc <- relevel(cData$phaseInc, ref = "testAB-B")

cData$phaseInc <- relevel(cData$phaseInc, ref = "testB-B")

cData$phaseInc <- relevel(cData$phaseInc, ref = "testA-B")

cData$phaseInc <- relevel(cData$phaseInc, ref = "testAB-A")

cData$phaseInc <- relevel(cData$phaseInc, ref = "testB-A")

cData$phaseInc <- relevel(cData$phaseInc, ref = "testA-A")

cData$phaseInc <- relevel(cData$phaseInc, ref = "searchAB")

cData$phaseInc <- relevel(cData$phaseInc, ref = "searchB")

cData$phaseInc <- relevel(cData$phaseInc, ref = "searchA")

cData$phaseInc <- relevel(cData$phaseInc, ref = "learning")

bpc<-ggplot(cData, aes(phaseInc, timeTo20, fill=trialPhase)) +

geom_boxplot() +

labs(title="Disruptive vs Distractive")+

scale_x_discrete(labels=c("Learning", "Drp", "Dac","Drp/Dac","Drp->Drp","Dac->Drp","Drp/Dac->Drp","Drp->Dac","Dac->Dac","Drp/Dac->Dac")) +

labs(y=" ")+

labs(x="Trial Phase")+

scale_y_continuous(trans = "log", breaks = c(800,1600,3200,6400,12800)) +

theme(panel.grid.major = element_blank(), panel.grid.minor = element_blank(), panel.background = element_blank(), panel.border = element_rect(colour = "black", fill=NA), axis.text.x=element_text(angle=-45, hjust = 0))

grid.arrange(bpa,bpb,bpc, ncol=1)
